# Salvage-Laryngektomie nach primärer Radio- und Radiochemotherapie: Eine retrospektive Fallzusammenfassung

**DOI:** 10.1007/s00106-021-01029-w

**Published:** 2021-04-09

**Authors:** Matti Sievert, Miguel Goncalves, Benedicta Binder, Sarina K. Mueller, Robin Rupp, Michael Koch, Stephan Dürr, Maximilian Traxdorf, Markus Hecht, Heinrich Iro, Antoniu-Oreste Gostian

**Affiliations:** 1 Hals-Nasen-Ohrenklinik, Kopf- und Hals-Chirurgie, Universitätskliniken Erlangen, Friedrich-Alexander-Universität Erlangen-Nürnberg, Waldstraße 1, 91054 Erlangen, Deutschland; 2 Strahlenklinik, Universitätskliniken Erlangen, Friedrich-Alexander-Universität Erlangen-Nürnberg, Erlangen, Deutschland

**Keywords:** Rezidiv, Salvage-Therapie, Larynxkarzinom, Therapie-Outcome, Postoperative Komplikationen, Recurrence, Salvage therapy, Laryngeal neoplasms, Treatment outcome, Postoperative complications

## Abstract

**Hintergrund:**

Das rezidivierende und residuelle Larynxkarzinom nach organerhaltender Radio- bzw. Radiochemotherapie ist mit einer schlechten Prognose verbunden. Die Salvage-Operation stellt in diesen Fällen die wichtigste therapeutische Option dar.

**Ziel der Arbeit:**

Erfasst wurden die Rate an Rezidiv- und Residualtumoren sowie die Überlebensraten und die Komplikationsrate nach Salvage-Chirurgie des Kehlkopfs an dem akademischen Tumorzentrum der Autor(inn)en.

**Material und Methoden:**

Retrospektiv wurden alle Patienten untersucht, bei denen zwischen 2001 und 2019 eine Salvage-Operation aufgrund eines Tumorresiduums oder Rezidivs nach primärer nichtchirurgischer Therapie erfolgt war.

**Ergebnisse:**

Es wurden 33 Salvage-Operationen durchgeführt. Die Defektrekonstruktion erfolgte in 30,3 % der Fälle (*n* = 10) mittels freier und in 15,2 % (*n* = 5) mittels regionaler Lappenplastik. Ein Patient hat sowohl eine freie als auch gleichzeitig eine gestielte Lappenplastik erhalten. Das Gesamtüberleben nach einem, 2 und 5 Jahren betrug 68,7 %; 47,9 % bzw. 24,2 %, das krankheitsfreie Überleben 81,6 %; 47,8 % bzw. 24,2 % bei insgesamt 48,5 % (*n* = 16) postoperativen Tumorrezidiven. Das krankheitsfreie Überleben war signifikant kürzer bei Tumorausdehnung im bzw. auf den Hypopharynx (*p* = 0,041). Postoperativ entwickelten 72,7 % der Patienten eine pharyngokutane Fistel unabhängig von einer simultanen Defektrekonstruktion. Nur 24 % der der aufgetretenen Fisteln mussten operativ therapiert werden. Der Krankenhausaufenthalt betrug 28,0 ± 16,1 Tage.

**Schlussfolgerung:**

Die Salvage-Laryngektomie ist mit vielen, aber beherrschbaren Komplikationen und einer hohen Morbidität verbunden. In Anbetracht der behandelten fortgeschrittenen Tumorkategorien und der Gesamtsituation des Patienten sind respektable onkologische Ergebnisse zu erreichen.

**Zusatzmaterial online:**

Die Online-Version dieses Beitrags (10.1007/s00106-021-01029-w) enthält eine Übersicht über die Patientenkohorte. Beitrag und Zusatzmaterial stehen Ihnen auf www.springermedizin.de zur Verfügung. Bitte geben Sie dort den Beitragstitel in die Suche ein, das Zusatzmaterial finden Sie beim Beitrag unter „Ergänzende Inhalte“.

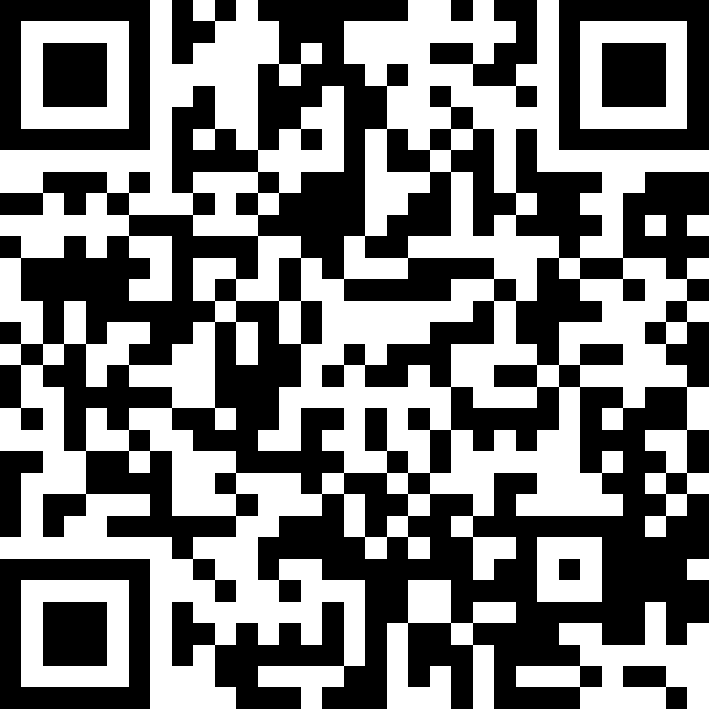

## Abstract (en)

**Background:**

Recurrent and residual laryngeal cancer after organ-preserving radio- or radiochemotherapy is associated with a poor prognosis. Salvage surgery is the most important therapeutic option in these cases.

**Objective:**

The study assessed rates of recurrence and residual tumor as well as survival and complication rates after salvage laryngectomy at the authors’ academic cancer center.

**Materials and methods:**

A retrospective examination of all patients receiving laryngectomy between 2001 and 2019 due to tumor residuals or recurrence after primary radio- and radiochemotherapy was conducted.

**Results:**

A total of 33 salvage procedures were performed. Defect reconstruction was performed by free flap surgery in 30.3% (*n* = 10) and regional flap surgery in 15.2% (*n* = 5) . One patient received regional flap surgery and free flap surgery simultaneously. Overall survival after 1, 2, and 5 years was 68.7, 47.9, and 24.2%, and disease-free survival was 81.6, 47.8, and 24.2%, respectively, with 48.5% (*n* = 16) postoperative tumor recurrences overall. Disease-free survival was significantly shorter for tumor extension into or onto the hypopharynx (*p* = 0.041). Postoperatively, 72.7% of patients developed a pharyngocutaneous fistula, of which 24.2% required surgical treatment. The hospital stay was 28.0 ± 16.1 days.

**Conclusion:**

Salvage laryngectomy is associated with a high rate of treatable complications and high morbidity. Nevertheless, considering the advanced tumor stages treated, it allows for respectable oncological results.

Die Salvage-Laryngektomie ist eine mögliche Therapieoption in der Behandlung des rezidivierenden und residuellen Larynxkarzinoms nach erfolgter Radio- oder Radiochemotherapie. Unter ständiger Konkurrenz und der rapiden Weiterentwicklung nichtoperativer Behandlungsmethoden des Kopf-Hals-Malignoms in den letzten Jahren ist die Erfassung der onkologischen Ergebnisse und der Komplikationen der Salvage-Chirurgie essenziell für die klinische Entscheidungsfindung.

## Hintergrund

Trotz zunehmender Tendenz zu primär organerhaltenden Ansätzen bei lokal fortgeschrittenen Plattenepithelkarzinomen des Larynx und des Hypopharynx kommt der „Rettungschirurgie“ (Salvage-Chirurgie) weiterhin eine große Bedeutung zu. Abhängig von der Lokalisation und dem Stadium des Primärtumors im Kopf-Hals-Bereich liegen die Rezidivraten nach primär organerhaltender Therapie bei 25–50 % [1]. In der Behandlung des lokoregionären Rezidivs ist eine wiederholte Strahlentherapie häufig ausgeschlossen, sodass mit der Rettungschirurgie effektive onkologische Ergebnisse erzielt werden können [2]. Die Wundheilung nach Strahlen- und Chemotherapie ist durch die Fibrosierung des Gewebes und eine Verminderung der Perfusion beeinträchtigt [3, 4]. Daraus ergibt sich eine deutlich erhöhte Komplikationsrate von bis zu 60 %, verbunden mit deutlich gesteigerter Mortalität und Morbidität sowie einer signifikant beeinträchtigten Lebensqualität [5]. Die Inkaufnahme der bekannten Nachteile und Risiken der Rettungschirurgie kann nur durch ein verbessertes Überleben gerechtfertigt werden. Als Alternative zur Operation stehen nach Versagen der Erstlinientherapie heutzutage neben der palliativen Chemotherapie und der Wiederbestrahlung auch die Immuntherapie zur Verfügung [6]. Es gibt zahlreiche klinische Studien, die die Rolle von Checkpointinhibitoren sowohl als Einzelsubstanz als auch in Kombination mit etablierten Behandlungsmethoden untersuchen. Der aktuelle Paradigmenwechsel in der Therapie des rekurrenten fortgeschrittenen Larynxkarzinoms stellt erneut die Wertigkeit der Rettungschirurgie auf den Prüfstand. Das Ziel der vorliegenden Arbeit war die Bestimmung der Überlebensrate sowie die Bewertung präoperativer prognostischer Faktoren für das allgemeine und krankheitsfreie Überleben einer Rettungschirurgie von Residualtumoren oder Rezidiven anhand der an der Klinik der Autor(inn)en behandelten Patienten. Sekundäre Ziele umfassten die Auswertung chirurgischer und allgemeiner postoperativer Komplikationen und die Dauer der Sondenernährung.

## Studiendesign und Untersuchungsmethoden

Es handelt sich um eine retrospektive Kohortenstudie der Universitätsklinik Erlangen (Abteilung für Hals-Nasen-Ohrenheilkunde, Kopf- und Hals-Chirurgie). Die Studie wurde von der zuständigen Ethikkommission genehmigt (Votum 246_20 Bc) und in Einklang mit der Deklaration von Helsinki durchgeführt.

### Studiendesign

Die Analyse erfolgte mittels retrospektiver Auswertung von Patientenakten aus der Tumordatenbank der Klinik. Die ausgewählten Patienten haben wegen eines Larynxkarzinoms im Zeitraum von März 2001 bis Oktober 2019 in dem akademischen Tumorzentrum der Autor(inn)en eine totale oder partielle Laryngektomie erhalten. Einschlusskriterien waren ein Tumorresiduum oder ein Lokalrezidiv nach primär organerhaltender nichtchirurgischer Therapie. Als Residuum wurde ein verbleibender Tumoranteil definiert, welcher in einer Panendoskopie direkt nach erfolgter primärer Radio- oder Radiochemotherapie makroskopisch und histologisch bestätigt wurde. Als Rezidiv definierten die Autor(inn)en ein Wiederauftreten des Tumors nach einem freien Intervall und einer unauffälligen Panendoskopie im Anschluss an die Radio- oder Radiochemotherapie. Patienten, die sich im Erkrankungsstadium der Fernmetastasierung befanden oder inoperable Tumoren aufwiesen, wurden aus der Studie ausgeschlossen. Nach Exstirpation des Larynx bzw. Teillaryngektomie mit oder ohne begleitender Pharyngektomie wurden die Defekte je nach Größe primär verschlossen oder mit regionalen oder freien mikrovaskulären Lappenplastiken rekonstruiert. Die Anlage einer perkutanen endoskopischen Gastrostomie (PEG) erfolgte als prophylaktische Maßnahme. Eine Gastrografin-Ösophagographie wurde nach 10 Tagen postoperativ durchgeführt. Die Autor(inn)en dokumentierten die onkologischen Parameter (TNM, R‑Status), postoperative Komplikationen und den Status gemäß ECOG (Eastern Cooperative Oncology Group). Die Klassifikation des Erkrankungsstadiums erfolgte anhand der 8. Version der UICC (Union Internationale Contre le Cancer [7]).

### Zielparameter

Primäre Endpunkte der Studie waren die onkologischen Ergebnisse mit der Rate an lokalen und regionären Rezidiven und Fernmetastasen sowie das krankheitsfreie Überleben und das Gesamtüberleben. Die Überlebenszeit wurde vom Tag der Operation bis zum Todestag aufgrund einer beliebigen Ursache (Gesamtüberleben), dem Auftreten eines Rezidivs (krankheitsfreies Überleben), oder des Datums, an dem der Patient zuletzt als lebend (Gesamtüberleben und krankheitsfreies Überleben) oder als nicht krankheitsbedingt tot (krankheitsfreies Überleben) bekannt war, definiert. Die Autor(inn)en zensierten Patienten, die zum Zeitpunkt der Auswertung noch am Leben waren. Sekundäre Endpunkte stellten die Rate an postoperativen Komplikationen, die Dauer der Sondenernährung über die PEG und die Art der Ernährung bei der letzten Nachsorge dar.

### Statistische Auswertung

Die metrischen Parameter werden mit dem Mittelwert und der Standardabweichung (SD) angegeben. Die Häufigkeiten der Variablen werden in absoluten und relativen Werten (*n*; %) dargestellt. Die Überlebenskurven wurden mithilfe der Kaplan-Meier-Schätzung erstellt und anhand des Log-Rank-Tests verglichen. Assoziationen zwischen einzelnen anatomischen Lokalisationen mit dem Gesamtüberleben sowie dem krankheitsfreien Überleben wurden an univariaten Cox-Modellen getestet. Der Zusammenhang zwischen nominalen Variablen wurde mit dem χ^2^-Test geprüft. Ein *p*-Wert <0,05 wurde als statistisch signifikant angesehen. Für die statistische Auswertung verwendeten die Autor(inn)en IBM SPSS Statistics, Version 25.0 (Fa. IBM Corporation, Armonk/NY, USA).

## Ergebnisse

### Merkmale des Patientenkollektivs

Insgesamt wurden in dem angegebenen Zeitraum an dem Zentrum der Autor(inn)en 1327 Plattenepithelkarzinome des Larynx diagnostiziert. Bei 1134 Patienten erfolgte eine primär operative und bei 193 Patienten eine primär organerhaltende Therapie. Darunter erfüllten 33 Patienten die Einschlusskriterien (6 w., 27 m.; mittleres Alter: 63,9 ± 10 Jahre, Tab. 1). Die Erstlinientherapie erfolgte in 8 Fällen (24,2 %) mit einer intensitätsmodulierten Radiotherapie (bis 67,3 ± 3,9 Gy Gesamtdosis). In 25 Fällen (75,8 %) wurde eine simultane Radiochemotherapie (bis 69,4 ± 3,4 Gy Gesamtdosis; Cisplatin, 5‑Fluorouracil, Docetaxel). Bei 8 Patienten war eine Induktionstherapie vorausgegangen.

**Table 1:** 

*Geschlecht*	*n (%)*	
Männlich	27 (81,8)	
Weiblich	6 (18,2)	
**Variable**	**Initial**	**Salvage**
Alter	MW ± SD	MW ± SD
Jahre	61,2 ± 10	63,9 ± 10
*T‑Stadium*	*n (%)*	*n (%)*
T1	1 (3,0)	5 (15,1)
T2	9 (27,3)	4 (12,1)
T3	12 (36,4)	9 (27,3)
T4a	11 (33,3)	12 (36,4)
T4b	–	3 (9,1)
*N‑Stadium*	*n (%)*	*n* *=* *17 (%)*^*a*^
N0	16 (48,5)	15 (88,2)
N1	4 (12,1)	–
N2b	7 (21,2)	–
N2c	6 (18,2)	–
N3b	–	2 (11,8)
*UICC*	*n (%)*	*n (%)*
I	1 (3,0)	5 (15,2)
II	4 (12,2)	4 (12,1)
III	11 (33,3)	7 (21,1)
IVa	17 (51,5)	12 (36,4)
IVb	–	5 (15,2)
*Grading*	*n (%)*	*n* *=* *31 (%)*
G1	–	1 (3,2)
G2	15 (45,5)	10 (32,3)
G3	18 (54,5)	20 (64,5)
*ECOG*	*n* *=* *32 (%)*	*n (%)*
ECOG 0	9 (28,1)	4 (12,1)
ECOG 1	13 (40,7)	9 (27,3)
ECOG 2	8 (25,0)	15 (45,5)
ECOG 3	1 (3,1)	4 (12,1)
ECOG 4	1 (3,1)	1 (3,0)

*UICC* Union Internationale Contre le Cancer, *ECOG* Eastern Cooperative Oncology Group Performance Status, *MW* Mittelwert, *SD* Standardabweichung

^a^Bei 16 ist keine Neck-Dissection erfolgt

Im Fall eines Tumorresiduums (*n* = 12; 36,4 %) erfolgte die Salvage-Operation nach durchschnittlich 7,2 ± 3,8 Monaten. Patienten mit Lokalrezidiven (*n* = 20; 60,6 %) wurden durchschnittlich 25,4 ± 18,1 Monate nach Erstdiagnose operiert. Ein Patient (3 %) entwickelte nach einem tumorfreien Intervall von 27 Jahren ein Rezidiv. In Tab. 1 sind die Patienten- und Therapiecharakteristika zum Zeitpunkt der Erstdiagnose und bei der Salvage-Operation aufgeführt. Eine Übersicht über die Patientenkohorte ist im elektronischen Zusatzmaterial online zu finden.

### Salvage-Chirurgie

Die insgesamt 33 Salvage-Operationen erfolgten mittels kompletter (*n* = 31) oder partieller (*n* = 2) Laryngektomie. Tumorfreie Schnittränder (R0) konnten insgesamt in 30 Fällen (90,9 %) erreicht werden. Bei 3 Patienten (9,1 %) wurde eine R1-Situation vorgefunden. In 2 Fällen wurde der initial mittels eines intraoperativen Schnellschnitts diagnostizierte R0-Status durch den endgültigen pathologischen Befund als R1 deklariert. Bei einem Patienten wurde die Infiltration der prävertebralen Faszie intraoperativ festgestellt. Zeitgleich zur Salvage-Operation haben die Autor(inn)en in 17 Fällen (51,5 %) eine Neck-Dissection (Level II–V) durchgeführt (14 bilateral, 3 unilateral; Tab. 2). Ein positiver Halsstatus (ycN+) wurde präoperativ bei 6 Patienten (18,2 %) in der Computertomographie diagnostiziert und konnte in 2 Fällen (6,1 %) histopathologisch bestätigt werden. Die pathologische Reklassifizierung ergab: 5 (15,1 %) rpT1, 4 (12,1 %) rpT2, 9 (27,3 %) rpT3, 15 (45,5 %) rpT4 (Tab. 1). In 19 Fällen (57,6 %) erfolgte die Pharynxrekonstruktion mittels primärer Naht. Aufgrund größerer Resektionsdefekte wurde bei 10 Patienten (30,3 %) eine freie mikrovaskuläre Lappenplastik und bei 5 Patienten (15,2 %) eine regionale Lappenplastik mittels myokutanem Pektoralis-major-Lappen (*n* = 2) oder fasziokutanem Deltopektorallappen (*n* = 3) durchgeführt (Tab. 2). Davon erhielt ein Patient sowohl eine freie als auch eine gestielte Lappenplastik. Bei 28 Patienten (84,8 %) erfolgte im Rahmen der Therapie eine PEG-Versorgung (Tab. 3). Um eine Stimmrehabilitation zu erreichen, wurde bei 6 Patienten (18,2 %) eine primäre und bei einem Patienten (3,0 %) eine sekundäre tracheoösophageale Punktion mit Anlage einer Stimmprothese (Provox 1, 2 und Vega; Fa. Atos Medical, Malmö, Schweden) durchgeführt.

**Table 2:** 

**Primäre Therapie**	***n***	**%**
*Radiotherapie*	8	24,2
*Radiochemotherapie*^*a*^	25	75,8
*Induktion*^*b*^	8	24,2
**Bestrahlungsdosis (Gy)**	**MW**	**SD**
*Tumorregion*	68,9	3,6
*Lymphabflussgebiet*	58,2	6,2
**Zeitspanne (Bestrahlung – Op.)**	**MW**	**SD**
*Monate*	18,6	16,9
**Indikation zur Salvage-Laryngektomie**	***n***	**%**
*Residuum*	12	36,4
*Rezidiv*	20	60,6
*Zweitmalignom*	1	3,0
**Resektionsstatus**	***n***	**%**
*R0*	30	90,9
*R1*	3	9,1
**Pharynxrekonstruktion**	***n***	**%**
*Primäre Pharynxnaht*	19	57,6
*Gestielter Lappen*^*c,d*^	5	15,2
*Freier Lappen*^*e*^	10	30,3
**Neck-Dissection**	***n***	**%**
*Unilateral*	3	9,1
*Bilateral*	14	42,4

*MW* Mittelwert, *SD* Standardabweichung

^a^Simultane Radiochemotherapie

^b^Induktionschemotherapie (Cisplatin, 5‑Fluorouracil [5-FU], Docetaxel)

^c^Patient Nr. 15 wurde zur Rekonstruktion des Pharynx mit einem freien und einem gestielten Transplantat versorgt

^d^Davon 3 Deltopektorallappen und 2 Pektoralis-major-Lappen

^e^Davon 4 anterolaterale Oberschenkellappen und 6 Radialislappen

### Onkologische Ergebnisse

**Figure 1:**
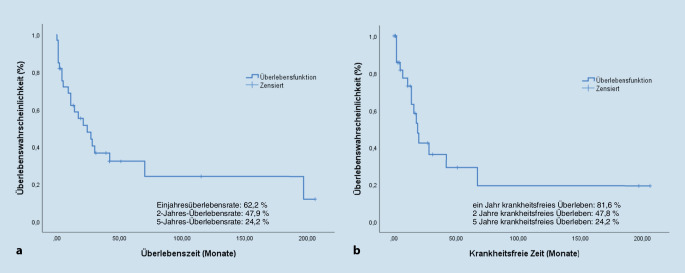

Insgesamt wiesen 16 der 33 Patienten (48,5 %) ein Tumorrezidiv nach der Salvage-Operation auf. Bei 9 Patienten (27,3 %) wurde ein Lokalrezidiv nach durchschnittlich 83,4 ± 87,8 Wochen und bei 3 Patienten (9,1 %) ein regionäres, zervikales Rezidiv nach 159,6 ± 121,2 Wochen festgestellt. Fernmetastasen wurden bei 11 Patienten (33,3 %) nach durchschnittlich 76,36 ± 80,6 Wochen diagnostiziert. Die Ein-, 2‑ und 5‑Jahres-Gesamtüberlebensrate betrug 68,7 %, 47,9 % bzw. 24,2 % mit einer medianen Überlebenszeit von 11 Monaten (0–206 Monate). Das krankheitsfreie Überleben nach einem, 2 und 5 Jahren betrug 81,6 %, 47,8 % bzw. 24,2 % (Abb. 1). In der univariaten Regressionsanalyse erwies sich die Tumormanifestation im Hypopharynx (*p* = 0,033) als signifikanter negativer Prädiktor für das Gesamtüberleben. Das krankheitsfreie Überleben war signifikant reduziert im Fall einer Tumormanifestation im Hypopharynx (*p* = 0,041).

### Postoperativer Verlauf

Eine Übersicht über alle chirurgischen und medizinischen postoperativen Komplikationen gibt Tab. 3. Komplikationen traten insgesamt bei 25 Patienten (75,7 %) auf. Die pharyngokutane Fistel war mit 72,7 % (*n* = 24) die häufigste Komplikation. In 16 Fällen (48,5 %) verschloss sich die Fistel nach rein konservativer Behandlung nach durchschnittlich 46,9 ± 35,6 Tagen. Bei 8 Patienten (24,2 %) wurde ein operativer Fistelverschluss mittels Pektoralis-major- oder Deltopektorallappen erforderlich. Die Autor(inn)en konnten keinen Zusammenhang zwischen Patienten mit und ohne primäre Lappenrekonstruktion in Bezug auf die Fistelrate feststellen (*p* = 0,618). Der Patient mit sekundärer Anlage einer Provox-Prothese entwickelte eine ausgedehnte ösophagotracheale Fistel. Die Patienten waren durchschnittlich 28,0 ± 16,1 in stationärer Betreuung, davon 6,0 ± 5,8 Tage auf der Intensivstation. Bei der letzten Nachsorgeuntersuchung nach durchschnittlich 34,0 ± 50,8 Monaten ernährten sich 16 Patienten (50,0 %) vollständig oral. Die PEG wurde bei diesen Patienten nach durchschnittlich 39,7 ± 20,8 Wochen entfernt. Bei der letzten Vorstellung waren 5 Patienten (15,6 %) teilweise und 11 Patienten (34,4 %) vollständig auf die PEG angewiesen (Tab. 3).

**Table 3:** 

*Chirurgische Komplikationen*	*n (%)*	*Dauer in Tagen (MW* *±* *SD)*
Fistel ohne chirurg. Revision	16 (48,5)	46,9 ± 35,6
Fistel mit chirurg. Revision	8 (24,2)	201,0 ± 170,9
Ösophagotracheale Fistel	1 (3,0)	–
Wundheilungsstörungen	14 (42,4)	–
*Medizinische Komplikationen*	*n (%)*	*–*
Lungenarterienembolie	1 (3,0)	
Schlaganfall	2 (6,1)	
*Dauer des stationären Aufenthalts*	*–*	*Dauer in Tagen (MW* *±* *SD)*
		28,0 ± 16,1
*Dauer des Aufenthalts auf Intensivstation*	*–*	*Dauer in Tagen (MW* *±* *SD)*
		6,0 ± 5,8
*PEG-Versorgung*	*n (%)*	*Dauer in Wochen (MW* *±* *SD)*
Gesamt	28 (84,8)	39,7 ± 20,8
Vor Salvage-Operation	19 (57,6)	
Bei Salvage-Operation	8 (24,2)	
Nach Salvage-Operation	1 (3,0)	
*Ernährung bei letzter Vorstellung*	*n* *=* *32 (%)*	*–*
Komplett über PEG-Sonde	11 (34,4)	
Teilweise über PEG-Sonde	5 (15,6)	
Normale oder weiche Kost (d. h. PEG entfernt)	16 (50,0)	

*PEG* perkutane endoskopische Gastrostomie, *MW* Mittelwert, *SD* Standardabweichung

## Diskussion

In der vorliegenden Arbeit wurde der Verlauf von Patienten nach Rettungschirurgie durch eine totale Laryngektomie oder Teillaryngektomie nach primär nichtchirurgischer Therapie untersucht. Die vorliegenden Ergebnisse zeigen ein 2‑ und ein 5‑Jahres-Gesamtüberleben von 47,9 bzw. 24,2 % (Abb. 1) und ein krankheitsfreies Überleben von 47,8 bzw. 24,2 %. Insgesamt wiesen 48,5 % der Patienten postoperativ ein Tumorrezidiv auf. Die Autor(inn)en stellten eine signifikant schlechtere 5‑Jahres-Überlebensrate fest, wenn eine hypopharyngeale Beteiligung vorlag (28,8 vs. 10,9 %; *p* = 0,041). Die Rettungschirurgie war mit einer hohen Komplikationsrate verbunden. Dabei war die pharyngokutane Fistel die häufigste Komplikation, die zumeist lediglich einer konventionellen Therapie bedurfte. Die Hälfte der behandelten Patienten konnte sich postoperativ komplett oral ernähren.

Allgemein liegt die Rate der lokoregionären Rezidive nach organerhaltender Therapie bei 30–50 % [1, 8]. Besteht nach primärer Radiochemotherapie ein Tumorresiduum oder ein lokoregionäres Rezidiv, sollte die Möglichkeit der Salvage-Chirurgie geprüft werden. Sie stellt nach Versagen der primär nichtchirurgischen Therapie weiterhin eine kurative Therapieform dar, sofern eine vollständige Resektion mit negativen Rändern präoperativ erreichbar erscheint. Über 70 % der Patienten mit einem Residuum oder Rezidiven nach Radiochemotherapie weisen lokal fortgeschrittene Tumoren der Kategorien T3 und T4 auf [9]. Dies können die Autor(inn)en mit 27,3 % der Patienten in einem frühen (UICC I und II) und 72,7 % der Patienten in einem lokal fortgeschrittenen Tumorstadium (UICC III und IV) bestätigen. In Anbetracht des Untersuchungszeitraums und der darin begründeten Entwicklung der Bestrahlungstechnik sind die von den Autor(inn)en mittels Rettungschirurgie behandelten Patienten mit den Angaben von Putten et al. [5] für die Untersuchungsjahre 1990–2007 vergleichbar. Damit bleibt die Rettungschirurgie für den onkologischen Chirurgen weiterhin eine unverändert anspruchsvolle und komplikationsbehaftete chirurgische Therapiemöglichkeit.

Die onkologischen Ergebnisse sind therapieübergreifend insgesamt unbefriedigend. Für rein laryngeale Karzinome wird zusammengefasst ein Gesamtüberleben von 48–49 % und mit Beteiligung des Hypopharynx von 17–26 % angegeben [1, 10]. Alternativ kann eine erneute Radio- oder Radiochemotherapie in kurativer Absicht angeboten werden. Eine umfassende Metaanalyse zu den Ergebnissen der Wiederbestrahlung wurde von Grün et al. kürzlich veröffentlicht. Die Kollegen berichten über eine 2‑ und 5‑Jahres-Gesamtüberlebensrate von 47–57 % und 23 % nach intensitätsmodulierter Radiotherapie mit begleitender Chemotherapie [11]. Beachtlich sind die damit verbundenen, teilweise gravierenden Nebenwirkungen mit schwerwiegenden Akutreaktionen in bis zu 73 %. Akute lebensbedrohliche Komplikationen wurden in bis zu 11 % der Fälle beschrieben [11, 12]. Die alleinige palliative Therapie führte Kowalski et al. zufolge an 797 Patienten mit rezidivierendem Kopf-Hals-Malignomen zu einem medianen Überleben von nur 3,8 Monaten und ist bei möglicher Operabilität zurückhaltend zu empfehlen [13]. Eine erschwerte intraoperative Präparation durch narbige postradiogene Veränderungen und die Minderdurchblutung des Gewebes sind charakteristisch für eine Salvage-Operation [2]. Dies bedingt die deutlich erhöhten postoperativen Komplikationsraten mit Wundheilungsstörungen, Lymphödemen und v. a. der Ausbildung von pharyngokutanen Fisteln, die die häufigste chirurgische Komplikation darstellen [4].

Die konservative Therapie steht in solchen Fällen im Vordergrund, sodass nur bei rund einem Drittel aller Fisteln eine operative Versorgung notwendig ist [4, 14, 15]. Insbesondere Patienten mit einer revisionsbedürftigen Fistel zeigten einen prolongierten Heilungsverlauf mit deutlicher Einschränkung der Lebensqualität [15]. Die Rekonstruktion des Pharynx mit frischem Gewebe, bespielweise durch die Präparation eines Pektoralis-major-Lappens, hat einen protektiven Effekt auf die Fistelrate [15]. In Anbetracht der zu erwartenden Komplikationen nach der Salvage-Chirurgie und zum Erhalt einer akzeptablen Lebensqualität und Ernährung trägt auch die Anlage einer PEG bei [16, 17]. Zwei Drittel der Patienten waren bei der letzten Vorstellung, nach durchschnittlich 25 Monaten, nur teilweise oder gar nicht auf die Sondenernährung angewiesen.

Aufgrund der Problematik eines präoperativen „understaging“, bedingt durch postradiogene Ödeme und narbige Veränderungen, sowie der häufig multizentrischen Tumorherde der Rezidive ist die totale Laryngektomie weiterhin das präferierte Verfahren in der Salvage-Chirurgie des Larynxkarzinoms [10, 18, 19]. Die Ergebnisse dieser seltenen und anspruchsvollen Therapie müssen in Anbetracht des retrospektiven Studiendesigns und der damit unausweichlich verbundenen Limitationen interpretiert werden. Daraus resultiert ebenfalls die limitierte Patientenanzahl, die vergleichbaren Studien zu diesem Thema entspricht, jedoch die Analyse einzelner Einflussfaktoren auf die betrachteten onkologischen Ergebnisse beeinträchtigt. Ein weiterer Aspekt ist der große Zeitraum der retrospektiven Datenerhebung, der wirkungsvolle Entwicklungen der Radioonkologie umfasst. Dennoch sind die Charakteristika der zu behandelnden Patienten mit denen aus den 1990er- und ersten 2000er-Jahren vergleichbar.

Die Rettungschirurgie des Kehlkopfkarzinoms ist weiterhin eine Therapiemöglichkeit, die auch heutzutage noch ihren Stellenwert gegenüber alternativen Optionen der Wiederbestrahlung hat und in diesen anspruchsvollen Situationen mit dem Patienten ausführlich erörtert werden sollte.

## Fazit für die Praxis

- Die Salvage-Laryngektomie bleibt nach Organerhaltungsprotokollen die derzeit beste Therapieoption von Residuen und Rezidiven in kurativer Intention und sollte als Therapieoption in Betracht gezogen werden.
- Insbesondere Pateinten mit kleinen, rein laryngealen Tumoren ohne zervikale Metastasierung profitieren von einer Salvage-Laryngektomie.
- Komplikationen, v. a. pharyngokutane Fisteln, sind häufig, jedoch vorwiegend konservativ und selten chirurgisch erfolgreich zu therapieren.

## Supplementary Information




