# Supplementary material for: Salvage laryngectomy after primary radio- and radiochemotherapy: A retrospective study. German version
Source: HNO. 2021 Apr 9;70(1):44–50. [Article in German] doi: 10.1007/s00106-021-01029-w (PMC8760217; doi:10.1007/s00106-021-01029-w)
Supplement: Supplementary file 1 [file 106_2021_1029_MOESM1_ESM.pdf]

Übersicht über die Patientenkohorte

Nr.	G	A (ED)	TR	cT	cN	UICC (ED)	PT	TG (Gy)	yrcT	yrcN	OP	A (OP)	ND	Reko	rpT	rpN	UICC (OP)	R	Rez	tR (W)	Status	ÜZ (M)	KFÜ (M)
1	m	42	La	cT2	cN1	III	RCT	65,0	T2	N0	kLE	70	-	-	pT1		I	R0	-		L		2,0
2	w	67	HP	cT4a	cN2b	IV	RCT	72,0	T4a	N1	kLE	70	b	Ra	pT3	pN0	III	R0	-		L		3,0
3	m	59	La	cT3	cN2c	IV	RCT	66,0	T4a	N2b	kLE	59	b	ALT	pT3	pN3b	IV	R0	-		L		13,0
4	m	72	La	cT3	cN0	III	RCT	66,0	T3	N0	kLE	75	b	-	pT3	pN0	III	R0	-		T	4,0	4,0
5	w	51	La	cT4a	cN2b	IV	RT	68,0	T4a	N0	kLE	54	b	Ra	pT4a	pN0	IV	R0	l	82	L		18,0
6	m	55	La	cT3	cN0	III	RCT	72,0	T3	N0	kLE	56	-	-	pT4a		IV	R0	-		L		31,0
7	m	72	La	cT3	cN2b	IV	RCT	70,0	T3	N1	kLE	74	b	-	pT4b	pN0	IV	R0	f	12	T	4,0	2,0
8	m	55	La	cT2	cN2b	IV	RCT	72,0	T3	N0	kLE	56	b	-	pT3	pN3b	IV	R0	f	125	L		28,0
9	m	53	La	cT4a	cN0	IV	RCT	68,0	T4a	N0	kLE	55	-	-	pT3		III	R0	f	46	L		10,0
10	m	52	La	cT2	cN0	II	RCT	66,0	T3	N0	kLE	55	-	ALT	pT3		III	R0	l/r/f	62	L		14,0
11	m	62	HP	cT4a	cN2c	IV	RCT	70,0	T4a	N0	kLE	67	-	Ra	pT1		I	R0	f	88	T	28,0	20,0
12	m	49	La	cT2	cN2b	IV	RCT	72,0	T2	N0	kLE	49	b	-	pT2	pN0	II	R0	-		L		51,0
13	w	60	La	cT2	cN2b	IV	RCT	66,0	T2	N0	kLE	62	u	Ra	pT2	pN0	II	R0	f	33	T	42,0	7,0
14	m	82	La	cT4a	cN1	IV	RCT	72,0	T4a	N0	kLE	83	b	-	pT3	pN0	III	R0	f	11	T	2,0	2,0
15	m	69	HP	cT3	cN0	III	RCT	70,6	T4a	N0	kLE	69	b	Ra/DP	pT3	pN0	III	R0	-		T	1,0	1,0
16	m	60	HP	cT4a	cN2b	IV	RCT	70,0	T4a	N1	kLE	61	b	Ra	pT3	pN0	III	R0	r/f	61	T	14,0	14,0
17	w	65	HP	cT4a	cN0	IV	RCT	71,2	T3	N0	kLE	67	-	-	pT4a		IV	R1	-		T	1,0	0,0
18	m	58	HP	cT3	cN0	III	RCT	73,6	T3	N0	kLE	62	u	PM	pT4b	pN0	IV	R0	-		T	0,0	0,0
19	m	68	HP	cT2	cN0	II	RCT	72,0	T4a	N0	kLE	72	-	ALT	pT4a		IV	R0	-		T	1,0	1,0
20	w	54	HP	cT3	cN1	III	RCT	70,0	T4a	N0	kLE	55	u	ALT	pT4b	pN0	IV	R0	l	12	L		2,0
21	m	69	HP	cT2	cN2c	IV	RCT	71,8	T3	N0	kLE	71	-	PM	pT1		I	R0	-		T	27,0	27,0
22	m	49	HP	cT3	cN0	III	RCT	72,4	T3	N0	kLE	50	-	-	pT4a		IV	R0	l	85	T	24,0	19,0
23	m	62	La	cT4a	cN2c	IV	RCT	72,0	T4a	N1	kLE	63	b	-	pT4a	pN0	IV	R1	l	72	T	30,0	0,0
24	m	80	La	cT2	cN0	II	RT	70,2	T4a	N0	kLE	81	-	-	pT4a		IV	R0	l	49	T	11,0	11,0
25	m	86	La	cT1	cN0	I	RT	66,6	T2	N0	kLE	91	-	-	pT2		II	R0	-		T	11,0	11,0
26	m	78	La	cT3	cN0	III	RT	70,0	T4a	N0	kLE	81	-	DP	pT4a		IV	R1	-		T	5,0	0,0
27	m	68	La	cT3	cN1	III	RT	70,6	T4a	N0	kLE	68	-	DP	pT4a		IV	R0	l/f	22	T	9,0	5,0
28	m	51	HP	cT4a	cN0	IV	RCT	63,0	T4a	N0	kLE	54	b	-	pT4a	pN0	IV	R0	-		T	1,0	1,0
29	m	72	La	cT3	cN0	III	RT	70,2	T3	N0	kLE	72	-	-	pT4a		IV	R0	-		T	17,0	17,0
30	m	65	La	cT3	cN0	III	RT	64,0	T3	N0	kLE	70	-	-	pT4a		IV	R0	l/r/f	295	T	70,0	67,0
31	m	67	La	cT2	cN0	II	RT	59,4	T3	N0	kLE	69	b	-	pT2	pN0	II	R0	-		L		206,0
32	m	46	La	cT4a	cN2c	IV	RCT	60,0	T4a	N2b	KT	47	b	-	pT1	pN0	I	R0	-		T	197,0	197,0
33	w	63	La	cT4a	cN2c	IV	RCT	72,0	T4a	N0	KT	64	-	-	pT1		I	R0	l/f	11	T	21,0	2,0

Abkürzungen:

G: Geschlecht
A: Alter
ED: Erstdiagnose
TR: Tumorregion
La: Larynx
HP: Hypopharynx
cT: klinische Tumorausdehnung
cN: klinischer Befall der Lymphknoten
PT: Primärtherapie
TG: Tumorgebiet
RCT: Radiochemotherapie
RT: Radiotherapie
Gy: Gray
yrcT: klinische Tumorausdehnung nach Primärtherapie
yrcN: klinischer Befall der Lymphknoten nach Primärtherapie
kLE: Komplette Laryngektomie
KT: Kehlkopfteilresektion

ND: Neck Dissektion
b: bilateral (zweiseitig)
u: unilateral (einseitig)
Reko: Rekonstruktionsparameter
ALT: anterolateraler Oberschenkellappen
DP: Deltopectoral Lappen
PM: Pectoralis major Lappen
Ra: Radialis
rpT: histopathologische Tumorausdehnung Salvage OP
rpN: histopathologischer Lymphknotenbefall in Salvage OP
R: Resektionsstatus
Rez: Rezidiv
l: Lokalrezidiv
r: Regionalrezidiv
f: Fernmetastasen
L: Lebend
T: Tot

ÜZ: Überlebenszeit in Monaten
KFÜ: Krankheitsfreies Überleben in Monaten

Art der Fernmetastasen:

Nr. 7: mediastinaler Lymphknoten/pulmonal
Nr. 8: pulmonal
Nr. 9: pulmonal/Weichteilgewebe/ossär
Nr. 10: pulmonal
Nr. 11: pulmonal
Nr. 13: pulmonal
Nr. 14: pulmonal
Nr. 16: pulmonal/hepatisch/ossär
Nr. 27: Haut
Nr. 30: pulmonal
Nr. 33: pulmonal
